# Outcomes of modified trapeziectomy with ligament reconstruction tendon interposition for the treatment of advanced thumb carpometacarpal arthritis: Two-year follow-up

**DOI:** 10.1097/MD.0000000000010235

**Published:** 2018-03-30

**Authors:** Tao Wang, Gang Zhao, Yong-Jun Rui, Jing-Yi Mi

**Affiliations:** Department of Hand Surgery, Wuxi No. 9 People's Hospital Affiliated to Soochow University, Wuxi, China.

**Keywords:** flexor carpi radialis, modified, thumb carpometacarpal arthritis, trapeziectomy

## Abstract

Numerous arthroplasty techniques had been reported for the treatment of thumb carpometacarpal (CMC) joint osteoarthritis. The purpose of our study is to evaluate long-term clinical and radiographic outcomes of patients who underwent modified trapeziectomy with ligament reconstruction tendon interposition (LRTI).

Our retrospective study included 20 consecutive patients with advanced thumb CMC arthritis receiving modified trapeziectomy with LRTI (20 thumbs). For clinical evaluation, we assessed visual analogue scale (VAS), Disabilities of the Arm, Shoulder and Hand (DASH) scores and Kapandji index. Additionally, the grip, pinch power and waist flexion power, radial and volar abduction angle were evaluated, As for radiologic evaluation, we just estimated height of the trapezial space.

We took 2-year follow-up. All patients showed decreased VAS from 6.8 preoperatively to 1.4. Mean DASH and Kapandji scores were improved from 52.2 preoperatively to 21.6 and from 6.4 preoperatively to 7.4, respectively. Compared to preoperative range of motion (ROM) for radial abduction and volar abduction, both markedly increased at 2-year follow-up (from 61.2 to 80.1, from 60.6 to 78.3, respectively). Besides, mean power improved from 15.9 preoperatively to 21.7 kg at 2-year follow-up for grip power, from 1.9 preoperatively to 3.5 kg at 2-year follow-up for tip pinch; however, mean waist flexion power showed no significant change from 20.5 preoperatively to 19.7 kg at 2-year follow-up. Notably, there was no significant sinking in height of the trapezial space from 10.0 preoperatively to 9.6 mm at 2-year follow-up. NO case had a complication at final follow-up.

Modified trapeziectomy with LRTI treating thumb CMC arthritis in Eaton stage III–IV had a satisfactory efficacy. This new procedure is able to provides enough support for thumb to prevents thumb sinking.

## 1 Introduction

Previous studies^[1,2]^ reported that thumb carpometacarpal (CMC) arthritis is a common hand degenerative joint disease, occupying 11% and 33% of men and of women, respectively. Some authors believed that level of hormonal led to this difference of incidence.^[3]^ Thumb CMC arthritis could lead to pain, laxity, and weakness of the CMC joint.^[4]^ It is well known that thumb CMC joint is a biconcave–convex saddle joint including the first metacarpal of the thumb and the trapezium carpal bone. Above mentioned makes CMC joint motion in 3 different planes: adduction–abduction, flexion–extension, and axial rotation.^[5–7]^ There are 2 theories could explain the etiology of CMC arthritis. First, it is an evolutionary adaptation that human sacrifice stability at the basal joint in exchange of the ability to mobilize the thumb. Cartilage erosion and subsequent arthritis caused by instability and incongruity under long time of high-contact stress. Second, weakened palmar beak ligament maintaining the volar stability of the saddle joint increases high-contact stress between first metacarpal of the thumb and the trapezium carpal bone.^[8]^

The purposes of treatment for CMC arthritis are to reduce pain and preserve function of the joint. According to Eaton stage, as for patients with Eaton stage I, conservation therapy is better choice. However, as for progressing to Eaton stage II–IV, surgery is considered as the first selection.^[3–5]^ Badia^[9]^ conducted a retrospective study for patients with thumb CMC arthritis belonging to Eaton stage II and all patients received osteotomy. Final results confirmed that osteotomy was suitable for patients who had not complete articular cartilage loss. Avisar et al^[10]^ demonstrated that trapeziectomy with abductor pollicis longus (APL) arthroplasty gained a satisfactory efficacy for patients with thumb CMC arthritis in Eaton stage II–IV in long follow-up.

Increasing articles reported on trapeziectomy with ligament reconstruction tendon interposition (LRTI) treating thumb CMC arthritis. We retrospectively assessed the clinical and radiographic outcomes of modified trapeziectomy with LRTI in treatment for advanced thumb CMC arthritis at 2-year follow-up.

## 2 Materials and methods

### 2.1 Ethics statement

The study was approved by the Institutional Review Board of Wuxi No. 9 People's Hospital Affiliated to Soochow University before data collection and analysis.

### 2.2 Patients

We included 20 patients who underwent replantation from January 2014 to December 2015 in Wuxi No. 9 People's Hospital Affiliated to Soochow University. The inclusion criteria for study were as follows: belong to Eaton stage III or IV; more than 18 years old. The exclusion criteria were as follows: belong to Eaton stage I or II; have history of hand surgery; and younger than 18 years old.

### 2.3 Statistical analysis

**Figure 1:**
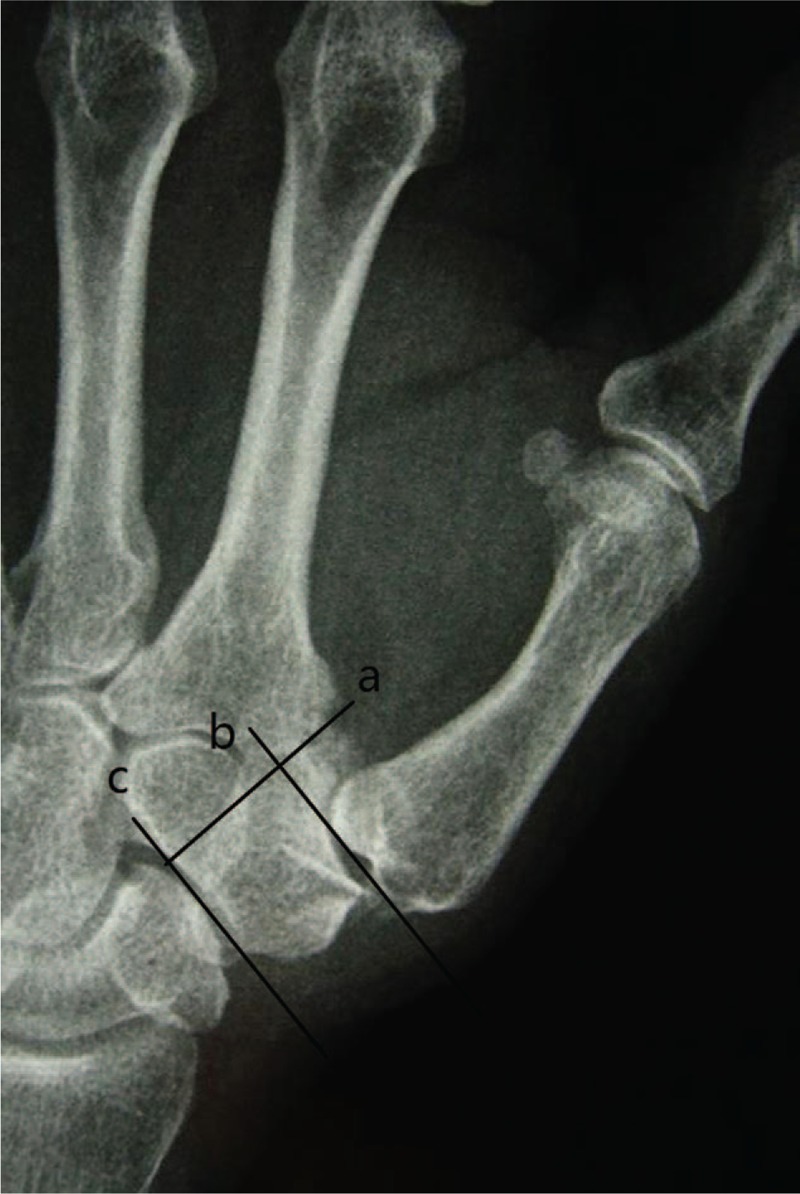
Hand X-ray showed that height of the trapezial space before surgery. Radiographic reference points for measurement of the height of the CMC space. Line (a) is the line projected through the radial articular surface of the index metacarpal the trapezium. Line (b) is the line tangent to the thumb metacarpal base and perpendicular to line (a). Line (c) is the line tangent to the distal extreme of the scaphoid and perpendicular to line (a). The distance between line (b) and line (c) is the height of the CMC space. CMC = carpometacarpal.

We assessed visual analog scale (VAS), Disabilities of the Arm, Shoulder and Hand (DASH), and Kapandji scores, grip, pinch power and waist flexion power, radial and volar abduction angle. As for radiologic evaluation, we just estimated height of the trapezial space. We measured preoperative and 2-year follow-up CMC space in the anteroposterior view as the distance between the distal scaphoid and the base of the metacarpal (Fig. 1). The methods were carried out in accordance with the approved guidelines. Two authors identified and collected all the data of patients according to inclusion criteria and exclusion criteria. In addition, 2 authors were responsible for data analyses. All measurement data were presented as the mean ± standard deviation when data satisfied criteria for normality with *P* > .05. When data like age, VAS, DASH, Kapandji, range of motion (ROM) of radial abduction and volar abduction, grip power, key-pinch, waist flexion power and height of the trapezial space satisfied criteria for normality and homogeneity of variance, statistical analysis between preoperation and final follow-up was performed using independent samples *t* test. The Kolmogorov–Smirnoff test was used to verify the normal data distribution. Statistical significance levels were considered to be *P* < .05. All statistical analyses were carried out using SPSS, version 21.0 (SPSS Inc, Chicago, IL).

### 2.4 Surgical technique

**Figure 2:**
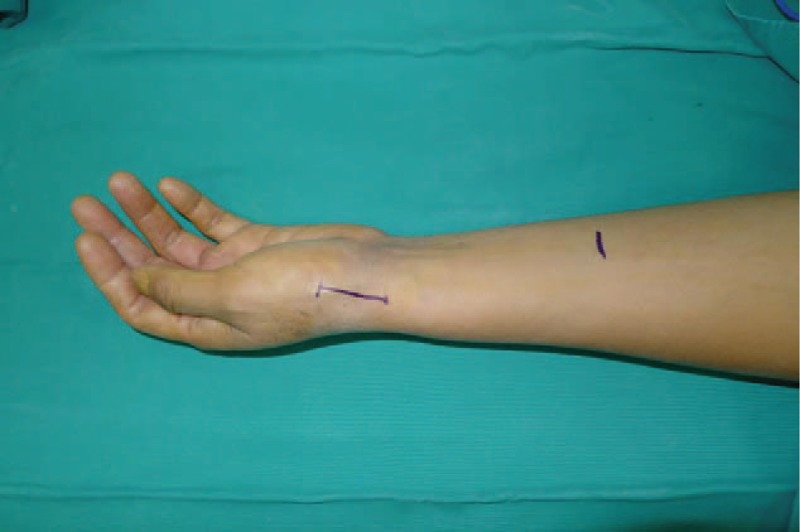
An incision is made at dorsal thumb metacarpal bone.

**Figure 3:**
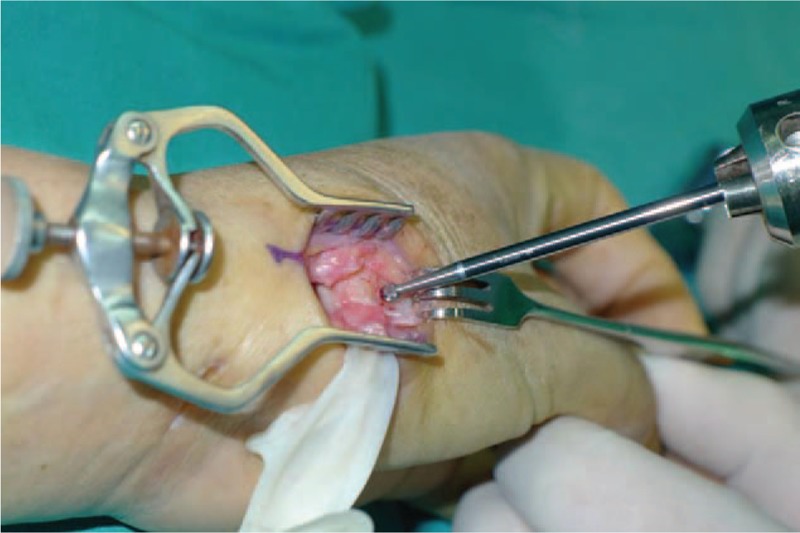
Expose trapezium and excised piecemeal.

**Figure 4:**
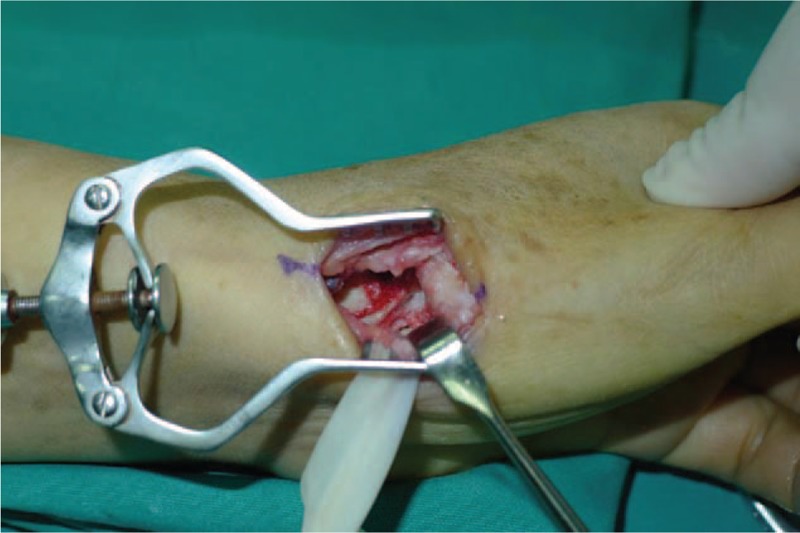
Remove trapezium.

**Figure 5:**
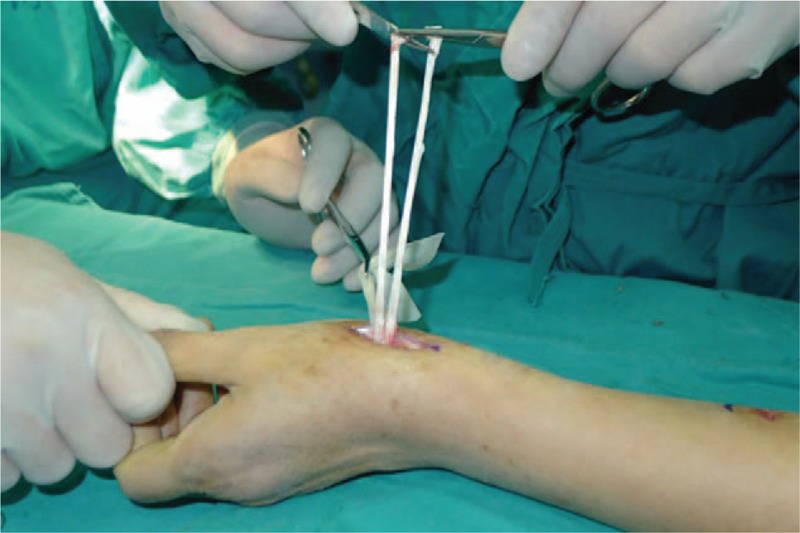
Cut flexor carpi radialis in half.

**Figure 6:**
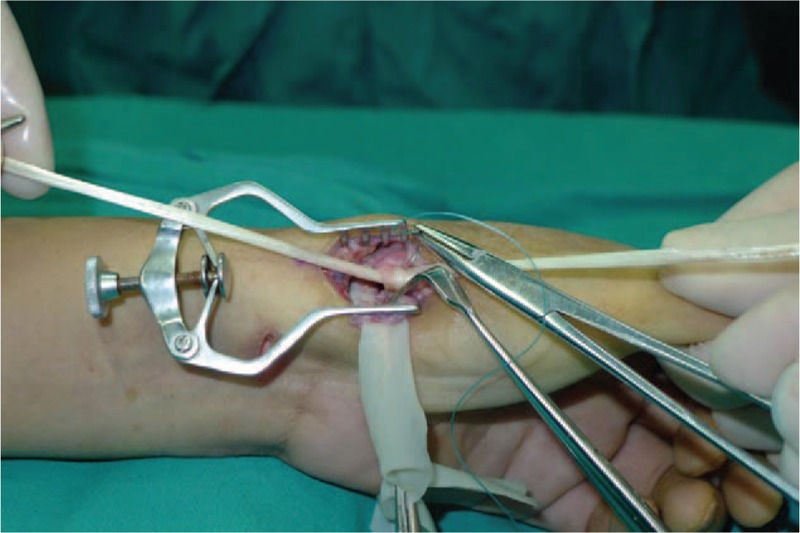
We passed one-half of flexor carpi radialis through the hole and tied with the other and sutured.

**Figure 7:**
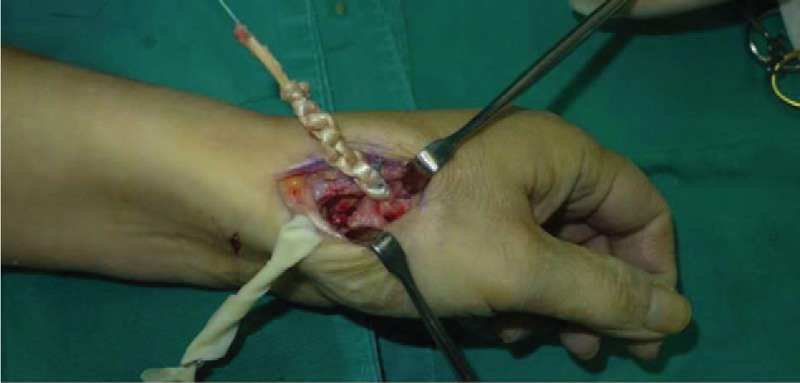
We sewed continuously and rolled flexor carpi radialis up.

**Figure 8:**
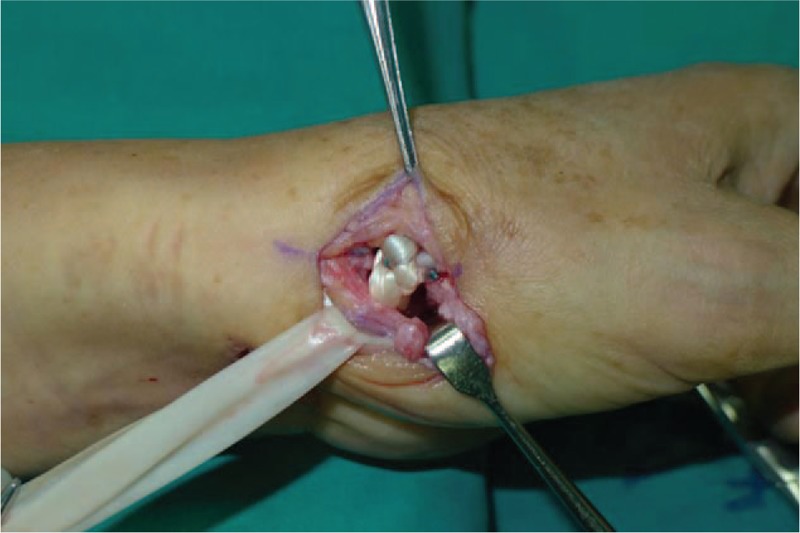
We put flexor carpi radialis into room where trapezium located in.

**Figure 9:**
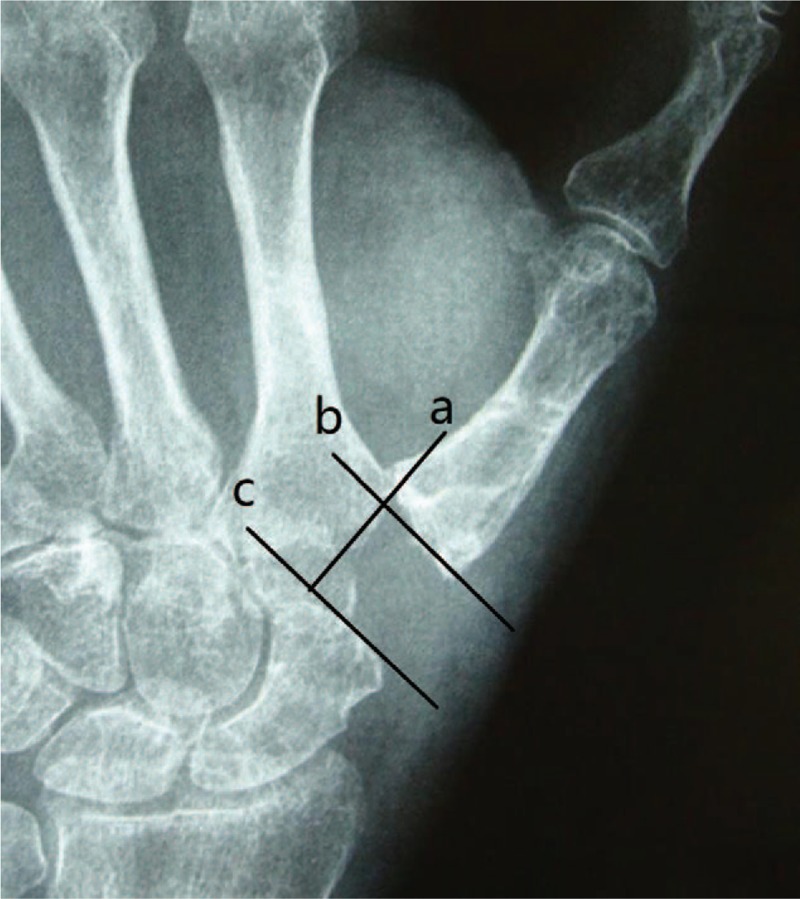
Hand X-ray showed that height of the trapezial space at 2-year follow-up. Radiographic reference points for measurement of the height of the CMC space. Line (a) is the line projected through the radial articular surface of the index metacarpal the trapezium. Line (b) is the line tangent to the thumb metacarpal base and perpendicular to line (a). Line (c) is the line tangent to the distal extreme of the scaphoid and perpendicular to line (a). The distance between line (b) and line (c) is the height of the CMC space. CMC = carpometacarpal.

All operations were performed by 1 surgeon. In Fig. 1, we could clearly see the height of the trapezial space is 10 mm and the thumb CMC arthritis belongs to Eaton stage III for X-ray. As shown in Fig. 2, we make a longitudinal incision at dorsal thumb metacarpal bone, afterwards a longitudinal incision was made for the capsule of the CMC, drawing attention to protecting superficial branch of radial nerve. After exposing trapezium, we remove it, then we could see flexor carpi radialis (FCR) tendon (Figs. 3 and 4). After drilling a hole from dorsal base to center of articular surface, we make a tiny incision in dorsal forearm and identified FCR. We cut whole FCR off at the transitive place between tendon and muscle and then pick it out from space created by trapeziectomy. As shown in Fig. 5, splitting longitudinally whole FCR into 2 halves, after that, we pass one-half through the hole and tie with the other and suture. Then the rest tendon is tied continuously, as shown in Fig. 6. We sew every knot and roll up like a ball (Fig. 7), then put the ball into space where previous trapezium locates in Fig. 8. We suture the tendon ball to volar capsule and ligament and close the capsule to prevent it escaping. Two years after surgery, the height of the trapezial space is 9.6 mm, as shown in Fig. 9.

### 2.5 Postoperative immobilization and rehabilitation

Gypsum fixation is conducted for a week and external fixation for 2 weeks. From 4th week, patients do functional exercise.

## 3 Results

Thirteen women and 7 men, whose mean age was 54.1 years old, were included in our study. There were 13 cases of stage III and 7 cases of stage IV arthritis (Table 1).

**Table 1:**
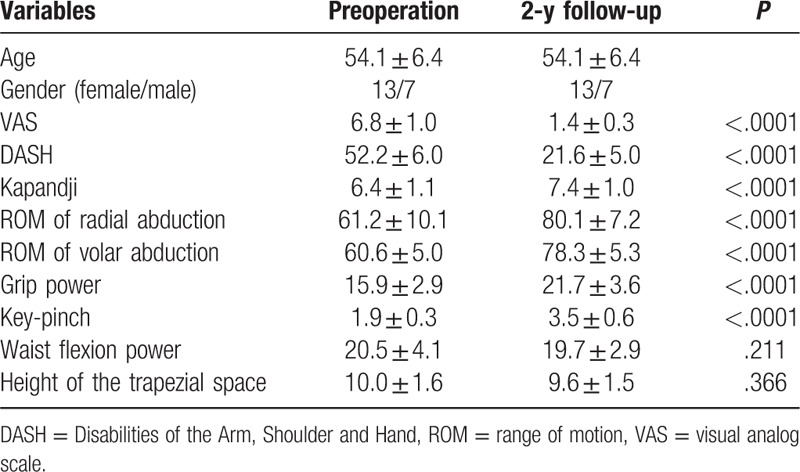
Preoperative and 2-year follow-up variables between 2 groups.

As shown in Table 1, mean VAS, DASH, and Kapandji score for thumb were 6.8, 52.2, and 6.4 before surgery, respectively. Preoperative mean ROM for radial abduction and volar abduction were 61.2° and 60.6°, respectively. Mean grip, key-pinch, and waist flexion power were 15.9, 1.9, and 20.5 kg preoperatively. Mean height of the trapezial space in hand X-ray was 10 mm. Two years after surgery, all patients expressed their satisfaction for improved appearance of the hand. VAS significantly declined to 1.4 points, additionally, DASH and Kapandji score improved to 21.6 and 7.4 points, respectively. Meanwhile, ROM for radial abduction and volar abduction obviously increased to 80.1° and 78.3°, respectively. Moreover, grip power was 21.7 kg and key-pinch power was 3.5 kg; however, waist flexion power was no significant reduction (from 20.5 to 19.7 kg) at final follow-up. It was worth noting that height of the trapezial space had no apparent sinking (from 10.0 to 9.6 mm; Table 1 and Figs. 1 and 9). No one had a complication at final follow-up.

## 4 Discussion

Thumb CMC arthritis is a commonly hand disease in hands. However, with regard to treat thumb CMC arthritis in Eaton stage II–IV, it remains controversial. The surgical procedure for thumb CMC joint includes not only simple trapeziectomy but also numerous ligament reconstruction surgeries. The simple trapeziectomy was the first reported by Gervis.^[11]^ In order to lower the risk of scaphometacarpal impingement caused by metacarpal subsidence, Froimson^[12]^ applied for autologous tendon interposed in the potential space created by the trapeziectomy. Kaarela and Raatikainen^[13]^ performed a retrospective study on outcomes of APL interposition arthroplasty and data showed that 76% got considerable pain relief; however, 68% of patients expressed that less thumb strength was less and 58% of patients had a slight or severe restriction to thumb ROM.

FCR, as a common tendon, is widely used in ligament reconstruction surgeries treating thumb CMC arthritis. Using half FCR in ligament reconstruction has been confirmed to stabilize the thumb basal joint and retard progression of osteoarthritis.^[14]^ But some authors doubted whether half of the FCR could provide enough adequate tension during reconstruction or not, which may be related with the tendon to slip.^[2]^ Saehle et al^[15]^ performed a comparison between APL and FCR as reconstructive tendon in the treatment of thumb CMC arthritis. He concluded that using FCR as reconstructive tendon had better outcomes in key-pinch and grip strengths. The aims of this article is to assess the clinical and radiographic outcomes of modified trapeziectomy with LRTI, ligament reconstruction with whole FCR, in treatment for advanced thumb CMC arthritis belonging to Eaton stage III–IV at 2-year follow-up.

In our study, we cut total FCR off and divided it into 2 parts and put one-half through the hole from dorsal base to center of articular surface at base of metacarpal bone to tie with the other, as shown in Figs. 5–7. Then we rolled it up and put it into room where previous trapezium located in (Fig. 8). Two years after surgery, VAS score for patients who underwent modified trapeziectomy with LRTI was outstanding reduction at final follow-up, indicating that pain has been relieved. While DASH and Kapandji score markedly improved, proving that hand function regained a satisfactory recovery. Besides, ROM for thumb in radial abduction and volar abduction markedly improved. What's more, we also had good results in grip and key-pinch power. Above data proved that use of whole FCR was able to effectively resolve pain and recover function.

We wondered whether using the entire FCR tendon significantly lower waist flexion power before surgery. However, the result dispelled our doubt, showing that the waist flexion power slightly weaken from 20.5 to 19.7 kg (*P* > 0.05). The first metacarpal sinking has always been a tough complication for treating thumb CMC arthritis. Chang and Chung^[16]^ explored the outcomes of patients receiving trapeziectomy and APL suspension arthroplasty and results showed a 32% loss in CMC height after surgery and an additional 11% proximal metacarpal migration 1 year after surgery. Lee et al^[17]^ evaluated the CMC height after APL suspension ligamentoplasty with 3 years follow-up and final data showed that the height of the space decreased from 10.8 mm preoperatively to 7.1 mm at final follow-up. In our study, it was notable that just 4% loss (from 10.0 to 9.6 mm) in CMC height was observed at 2 years after operation, as shown in Figs. 1 and 9. Compared to ligament reconstruction with APL as previous reported, entire FCR was able to provide greater support for thumb metacarpal to avoid subsidence. From the above, the data proved that modified trapeziectomy with LRTI using whole FCR was an effective treatment for thumb CMC arthritis and it did not markedly affect wrist flexion power.

We concluded following merits of this modified LRTI. First, according to anatomical knowledge, the end point of the FCR tendon is at the base of the second metacarpal, our method that one-half FCR tendon through the hole and tied with the other and sutured, as shown in Fig. 5, indirectly hold the thumb and index metacarpal together, offering an enough support to avoid first metacarpal subsidence. Second, the tendon-to-bone fashion fixation is able to stabilize first metacarpal and is also beneficial to recovery key-pinch power. Third, the method that one-half tied continuously and sutured with the other and tendon ball (Figs. 6 and 7) which is put it into room where previous trapezium located in provides adequate support for thumb metacarpal to delay sinking which causes short deformity and diminished strength.

Although this study provides several novel findings, it has some limitations. First, this is just a retrospective and single-center study. A big sample, prospective and multicenter study is needed; second, it is just short-term study. Long-term follow-up would be beneficial to evaluate efficacy, especially for observing whether there is a collision between the scaphoid and the metacarpal which may cause pain in the long term; the last but not the less, we did just assess clinical and radiological outcomes of ligament reconstruction with entire FCR, not did a comparison between modified trapeziectomy with LRTI and another procedures like modified trapeziectomy with LRTI using APL or half of the FCR. In further study, we would compare clinical and radiological outcomes between ligament reconstruction with entire FCR and another surgeries.

In conclusion, thumb CMC arthritis is a common disease in clinic. Increasing articles had been reported various surgeries treating thumb CMC arthritis. Modified trapeziectomy with LRTI using whole FCR is an effective treatment for advanced thumb CMC arthritis. This new procedure not only stabilizes thumb metacarpal, but also provides enough support for it, which slower its sinking. We provide a new method for surgeons when facing thumb CMC arthritis and we need further study to observe efficacy in longer-term follow-up.

## Author contributions

**Resources:** G. Zhao, J-Y. Mi.

**Writing – original draft:** T. Wang.

**Writing – review & editing:** T. Wang, Y-J. Rui.
